# Microglia and Astrocyte Activation by Toll-Like Receptor Ligands: Modulation by PPAR-*γ* Agonists

**DOI:** 10.1155/2008/453120

**Published:** 2008-06-19

**Authors:** Catherine Gurley, Jessica Nichols, Shuliang Liu, Nirmal K. Phulwani, Nilufer Esen, Tammy Kielian

**Affiliations:** Department of Neurobiology and Developmental Sciences, College of Medicine, University of Arkansas for Medical Sciences, Little Rock, AR 72205, USA

## Abstract

Microglia and astrocytes express numerous members of the Toll-like receptor (TLR) family that are pivotal for recognizing conserved microbial motifs expressed by a wide array of pathogens. Despite the critical role for TLRs in pathogen recognition, when dysregulated these pathways can also exacerbate CNS tissue destruction. Therefore, a critical balance must be achieved to elicit sufficient immunity to combat CNS infectious insults and downregulate these responses to avoid pathological tissue damage. We performed a comprehensive survey on the efficacy of various PPAR-*γ* agonists to modulate proinflammatory mediator release from primary microglia and astrocytes in response to numerous TLR ligands relevant to CNS infectious diseases. The results demonstrated differential abilities of select PPAR-*γ* agonists to modulate glial activation. For example, 15d-PGJ_2_ and pioglitazone were both effective at reducing IL-12 p40 release by TLR ligand-activated glia, whereas CXCL2 expression was either augmented or inhibited by 15d-PGJ_2_, effects that were dependent on the TLR ligand examined. Pioglitazone and troglitazone demonstrated opposing actions on microglial CCL2 production that were TLR ligand-dependent. Collectively, this information may be exploited to modulate the host immune response during CNS infections to maximize host immunity while minimizing inappropriate bystander tissue damage that is often characteristic of such diseases.

## 1. INTRODUCTION

Microglia and astrocytes participate in the genesis of innate immune responses in the CNS parenchyma [1, 2]. Their strategic placement at or near the blood-brain barrier likely makes both glial types sentinel cells for surveying pathogen entry in the CNS parenchyma. Indeed, both microglia and astrocytes are capable of producing a wide range of proinflammatory mediators in response to a diverse array of microbial stimuli [3, 4]. Therefore, it is likely that resident glia are pivotal for eliciting a local CNS inflammatory response through the initial production of inflammatory mediators, which in turn, leads to the recruitment of additional immune effector cells from the peripheral circulation.

Toll-like receptors (TLRs) are a group of pattern recognition receptors (PRRs) responsible for recognizing conserved motifs expressed on broad classes of microbes termed pathogen-associated molecular patterns (PAMPs) [5, 6]. Typically, PAMPs represent structural or nucleic acid motifs that are less likely to undergo mutation, ensuring efficient pathogen recognition with a limited receptor arsenal [7]. A total of 13 TLRs have been identified to date, each conferring recognition of conserved motifs from large subclasses of bacteria, viruses, yeast, and fungi [5–7]. In addition, recent evidence indicates that TLRs are also capable of sensing endogenous ligands produced during stress or injury referred to as danger-associated molecular patterns (DAMPs) that may participate in autoimmune induction [8–10]. Numerous TLRs have been identified on microglia and astrocytes and both glial types are responsive to numerous TLR ligands implicating their role in pathogen recognition (reviewed in [11, 12]). In addition, recent evidence links TLRs with the host response to CNS injury presumably via recognition of endogenous “danger signals” since classical microbial TLR ligands are not present (reviewed in [13–15]). Since TLRs have been implicated in both infectious and noninfectious diseases of the CNS (reviewed in [11, 13]), understanding their potential to influence the course of neuroinflammation is paramount and under certain conditions inappropriate TLR activation may contribute to excessive tissue destruction. Therefore, modulating TLR activity to achieve optimal benefit for the host may be a plausible strategy for minimizing tissue damage during neuroinflammatory disorders.

A group of compounds with reported anti-inflammatory effects in several models of inflammation, including the CNS, are ligands that interact with peroxisome proliferator-activated receptor-gamma (PPAR-*γ*) [16–18]. PPAR-*γ* is a member of the nuclear hormone receptor superfamily of ligand-activated transcription factors that regulate the expression of genes involved in reproduction, metabolism, development, and immune responses [17, 19]. A wide array of both natural and synthetic agonists for PPAR-*γ* have been identified including the naturally occurring prostaglandin metabolite 15d-PGJ2, thiazolidinediones (TZDs) a group of synthetic PPAR-*γ* agonists used for the treatment of diabetes, polyunsaturated fatty acids, and certain high affinity tyrosine derivatives. With regard to the CNS, several PPAR-*γ* agonists have been documented for their ability to attenuate both microglial and astrocyte activation in response to a diverse array of stimuli as well as impact the course of several neurodegenerative diseases [16, 18, 20–25]. Although we and others have demonstrated that select PPAR-*γ* agonists are potent inhibitors of TLR2 and TLR4 activation (PGN and LPS, resp.) [23, 24, 26–29], a comprehensive examination of the effects of PPAR-*γ* agonists on a wide array of TLR ligands is lacking. In addition, although several studies describing the responses of microglia and astrocytes to TLR ligands exist [30–34], no reports have systematically investigated the ability of PPAR-*γ* ligands to modulate glial activation in response to TLR signals. Therefore, the purpose of this study was to define the actions of a panel of PPAR-*γ* agonists on TLR ligand-induced activation of microglia and astrocytes. Although within the same class of compounds, not all PPAR-*γ* agonists shared similar regulatory properties in response to various TLR ligands. Indeed, in some cases, inflammatory mediator production was enhanced following PPAR-*γ* agonist treatment. Collectively, these results suggest selective actions of PPAR-*γ* agonists on glial responses to TLR ligands that could be exploited for specific neuroinflammatory/infectious conditions of the CNS.

## 2. MATERIALS AND METHODS

### 2.1. TLR ligands and PPAR-*γ* agonists

The following TLR agonists were used in this study (see Table 1) with the concentration of each and its TLR target identified in parenthesis: Pam3CSK4 (TLR2, 1 *μ*g/ml), polyinosine-polycytidylic acid (polyI:C; TLR3, 25 *μ*g/ml), lipopolysaccharide from *E. coli* O111:B4 (LPS; TLR4, 100 ng/ml), flagellin from *Salmonella typhimurium* (TLR5, 10 *μ*g/ml), single-stranded RNA (ssRNA; TLR7/8, 10 *μ*g/ml), and synthetic unmethylated CpG oligonucleotide (ODN; TLR9, 0.1 *μ*M and 5 *μ*M for microglia and astrocytes, resp.). All TLR ligands were obtained from InvivoGen (San Diego, Calif, USA).

The natural PPAR-*γ* agonist 15d-PGJ_2_ and synthetic TZDs ciglitazone, rosiglitazone, pioglitazone, and troglitazone were purchased from Cayman Chemical (see Table 2; PPAR-*γ* Pak; Ann Arbor , Mich, USA). Dose-response studies were performed for all TZDs (10, 30, and 100 *μ*M) and 15d- PGJ_2_ (5, 10, and 20 *μ*M) in both TLR-activated microglia and astrocytes.

### 2.2. Primary microglia and astrocyte cultures

Primary microglia and astrocytes were isolated from C57BL/6 pups (2 to 4 days of age) as previously described [35, 36]. The purity of glial cultures was evaluated by immunohistochemical staining using antibodies against CD11b (BD Pharmingen) and glial fibrillary acidic protein (GFAP, Invitrogen, Carlsbad, Calif, USA) to identify microglia and astrocytes, respectively. The purity of primary microglia and astrocyte cultures was approximately 98% and 95%, respectively.

Throughout this study, microglia and astrocytes were seeded into 96-well plates at 2 × 10^5^ or 1 × 10^5^ cells/well, respectively, and incubated overnight. The following day, glia were pretreated with various PPAR-*γ* agonists for 1 hour prior to stimulation with the TLR ligand panel for 24 hours. Cell-conditioned supernatants were collected at 24 hours following TLR ligand treatment for quantitation of proinflammatory mediator expression by ELISA.

### 2.3. Cell viability assays

The effects of PPAR-*γ* and TLR agonists on glial cell viability were evaluated using a standard MTT assay based upon the mitochondrial conversion of (3-[4,5-dimethylthiazol-2-yl]-2,5-diphenyltetrazolium bromide) (MTT) into formazan crystals. Results are reported as the raw OD_570_ values (mean ± SD).

### 2.4. Enzyme-linked immunosorbent assays (ELISAs)

Protein levels of TNF-*α* and CXCL2 (MIP-2) (Biosource) and IL-12 p40 and CCL2 (MCP-1, OptEIA, BD Pharmingen, San Jose, Calif, USA) were quantified in conditioned medium from PPAR-*γ* and TLR ligand-treated glia using ELISA kits according to the manufacturer's instructions (level of sensitivity = 15.6 pg/ml).

### 2.5. Nitrite assay

Nitrite levels, a stable end product resulting from the reaction of NO with molecular oxygen, were determined in astrocytes by adding 50 *μ*l of cell-conditioned culture medium with 50 *μ*l of Griess reagent (0.1% naphtyletylenediamine dihydrochloride, 1% sulfanilamide, 2.5% phosphoric acid, all from Sigma) in a 96-well plate. The absorbance at 550 nm was measured on a plate reader (Spectra Max 190, Molecular Devices, Sunnyvale , Calif , USA), and nitrite concentrations were calculated using a standard curve with sodium nitrite (NaNO_2_, Sigma, level of sensitivity, 0.4 *μ*M). Based on our previous findings where *S. aureus*-derived TLR ligands were potent inducers of NO in astrocytes but not microglia [24, 33, 34], we only quantitated NO levels in the former in the current study.

### 2.6. Statistics

Significant differences between experimental groups were determined by the Student's *t*-test at the 95% confidence interval using SigmaStat (SPSS Science, Chicago, Ill, USA).

In this study, we performed a minimum of two independent replicates of each experiment to confirm the results obtained. The reporting of our results as representative of “x” number of independent experiments was required since it is difficult to achieve identical levels of proinflammatory mediator expression with distinct preparations of primary glia. As a result, the absolute concentrations of the various proinflammatory mediators differed between individual experiments; however, the trends were consistent. This required us to report results from a single study where each experimental treatment was represented by 3-4 individual wells (i.e., biological replicates) and statistical analysis conducted.

## 3. RESULTS

### 3.1. Ability of PPAR-*γ* agonists to modulate microglial cytokine production in response to diverse TLR ligands

**Figure 1:**
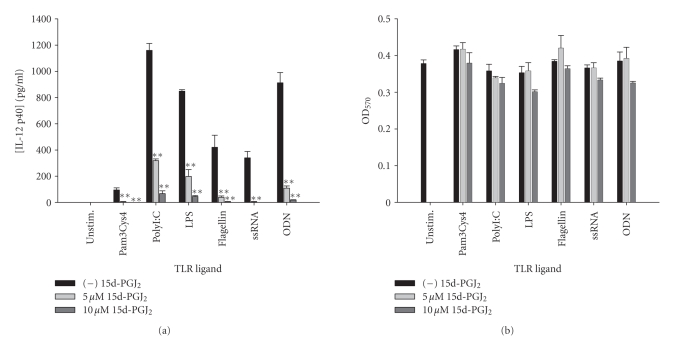
*The natural PPAR-*γ* agonist 15d-PGJ_2_ is a potent inhibitor of microglial IL-12 p40 production in response to a vast array of TLR ligands.* Primary microglia were plated at 2 × 10^5^ cells/well in 96-well plates and incubated overnight. The following day, cells were pretreated for 1 hour with the indicated concentrations of 15d-PGJ_2_ prior to the addition of TLR ligands. Cell-conditioned supernatants were collected at 24 hours following TLR ligand exposure, whereupon IL-12 p40 levels were determined by ELISA (a). The effects of 15d-PGJ_2_ on microglial viability were assessed using an MTT assay (b). Significant differences between microglia treated with TLR ligands alone versus TLR ligands + 15d-PGJ_2_ are noted with asterisks (**P < .001). Results presented are representative of two independent experiments.

**Figure 2:**
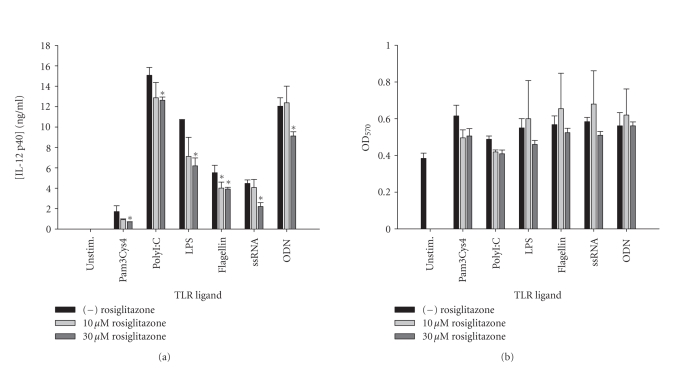
*The synthetic PPAR-*γ* agonist rosiglitazone attenuates IL-12 p40 production in response to TLR ligands in microglia.* Primary microglia were plated at 2 × 10^5^ cells/well in 96-well plates and incubated overnight. The following day, cells were pretreated for 1 hour with the indicated concentrations of rosiglitazone prior to the addition of TLR ligands. Cell-conditioned supernatants were collected at 24 hours following TLR ligand exposure, whereupon IL-12 p40 levels were determined by ELISA (a). The effects of rosiglitazone on microglial viability were assessed using an MTT assay (b). Significant differences between microglia treated with TLR ligands alone versus TLR ligands + rosiglitazone are noted with asterisks (* *P* < .05). Results presented are representative of two independent experiments.

**Figure 3:**
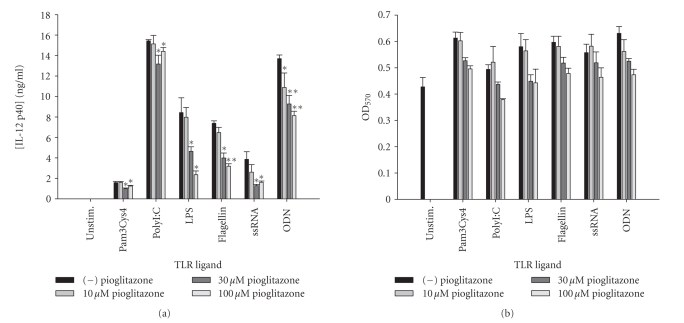
*The TZD pioglitazone inhibits microglial IL-12 p40 expression in response to diverse TLR ligands.* Primary microglia were plated at 2 × 10^5^ cells/well in 96-well plates and incubated overnight. The following day, cells were pretreated for 1 hour with the indicated concentrations of pioglitazone prior to the addition of TLR ligands. Cell-conditioned supernatants were collected at 24 hours following TLR ligand exposure, whereupon IL-12 p40 levels were determined by ELISA (a). The effects of pioglitazone on microglial viability were assessed using an MTT assay (b). Significant differences between microglia treated with TLR ligands alone versus TLR ligands + pioglitazone are noted with asterisks (* *P* < .05;**P < .001). Results presented are representative of two independent experiments.

Microglia represent the main innate immune effector in the CNS parenchyma as evident by their expression of numerous TLRs [32, 37, 38]. Although much emphasis has been placed on the neurodestructive properties of activated microglia, recent studies have revealed that in the correct context microglia can also facilitate CNS repair [39–41]. Therefore, striking the correct balance between regulated and inappropriate microglial activation may lead to optimal outcomes for a wide range of CNS neuroinflammatory conditions. To determine whether PPAR-*γ* agonists could serve to modulate microglial activation in the context of CNS infection, the effects of these compounds on microglial cytokine production in response to diverse TLR ligands were examined. The natural PPAR-*γ* agonist 15d-PGJ_2_ was uniformly found to inhibit IL-12 p40 release in response to all TLR ligands tested including Pam3Cys4, polyI:C, LPS, flagellin, ssRNA, and ODN (Figure 1). Fairly comprehensive reductions in IL-12 p40 production were also observed with all TLR ligands tested in response to the synthetic PPAR-*γ* agonists rosiglitazone (Figure 2) and pioglitazone (Figure 3) although the extent of inhibition was dramatically less compared to 15d-PGJ_2_. Rosiglitazone exhibited significant toxicity to primary microglia at the highest dose tested (i.e., 100 *μ*M), hence it was not included in the final analysis. In contrast, ciglitazone did not dramatically affect IL-12 p40 production in response to the majority of TLR ligands examined (data not shown). Troglitazone exhibited microglial toxicity at the two highest doses of agonist tested (i.e., 100 and 30 *μ*M); therefore, the results of this PPAR-*γ* agonist on microglial mediator production in response to TLR ligands are not presented.

**Figure 4:**
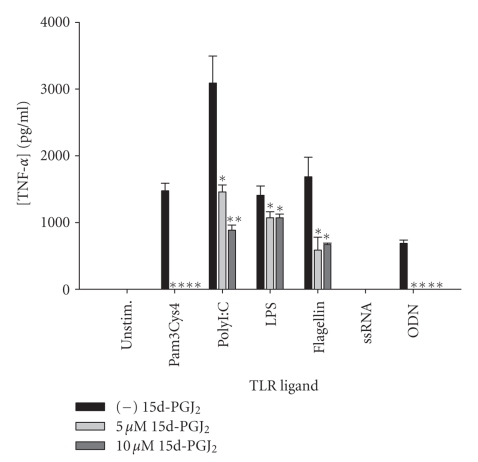
*The potency of 15d-PGJ_2_ to attenuate TNF-*α* production varies according to the TLR ligand examined.* Primary microglia were plated at 2 × 10^5^ cells/well in 96-well plates and incubated overnight. The following day, cells were pretreated for 1 hour with the indicated concentrations of 15d-PGJ_2_ prior to the addition of TLR ligands. Cell-conditioned supernatants were collected at 24 hours following TLR ligand exposure, whereupon TNF-*α* levels were determined by ELISA. Significant differences between microglia treated with TLR ligands alone versus TLR ligands + 15d-PGJ_2_ are noted with asterisks (*P < .05;**P < .001). Results presented are representative of two independent experiments.

**Figure 5:**
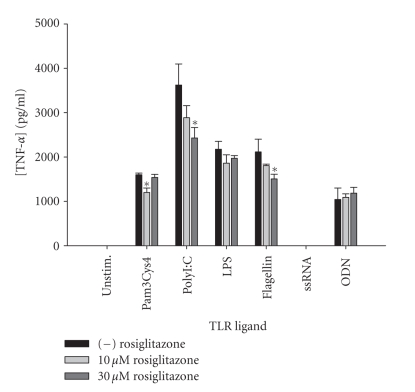
*The synthetic PPAR-*γ* agonist rosiglitazone selectively inhibits microglial TNF-*α* expression in response to TLR ligands.* Primary microglia were plated at 2 × 10^5^ cells/well in 96-well plates and incubated overnight. The following day, cells were pretreated for 1 hour with the indicated concentrations of rosiglitazone prior to the addition of TLR ligands. Cell-conditioned supernatants were collected at 24 hours following TLR ligand exposure, whereupon TNF-*α* levels were determined by ELISA. Significant differences between microglia treated with TLR ligands alone versus TLR ligands + rosiglitazone are noted with asterisks (*P < .05). Results presented are representative of two independent experiments.

Another proinflammatory cytokine with potent effects on the blood-brain barrier as well as glial activation is TNF-*α* [42]. This cytokine is expressed at high levels in numerous CNS infectious diseases including bacterial meningitis, brain abscess, as well as viral infections [43–46]. In some cases, excessive TNF-*α* production during these infectious diseases has been implicated in contributing to bystander damage to surrounding host tissue [45, 46]. Therefore, strategies aimed at achieving optimized cytokine expression may prove beneficial for favorable disease outcomes. Of the PPAR-*γ* agonists tested, 15d-PGJ_2_ was found to exert the most global inhibition of TNF-*α* production in response to the battery of TLR ligands tested (Figure 4). Specifically, TNF-*α* release by microglia in response to Pam3Cys4, polyI:C, flagellin, and ODN was significantly attenuated by 15d-PGJ_2_, whereas cytokine production following LPS treatment was not as dramatically affected. Single-stranded RNA (ssRNA) was a poor inducer of TNF-*α* by primary microglia (Figure 4). Similar to results with IL-12 p40, rosiglitazone was the next more global inhibitor of TNF-*α* production in response to the TLR ligands tested (Figure 5), whereas the other PPAR-*γ* agonists (i.e., ciglitazone and pioglitazone) were largely without effect (data not shown). Collectively, these results indicate that not all PPAR-*γ* agonists are equally effective at modulating proinflammatory cytokine release from primary microglia and suggest that tailored responses to specific pathogen motifs may be achieved through the use of distinct PPAR-*γ* agonists.

### 3.2. PPAR-*γ* agonists differentially affect chemokine release by microglia following TLR ligand treatment

**Figure 6:**
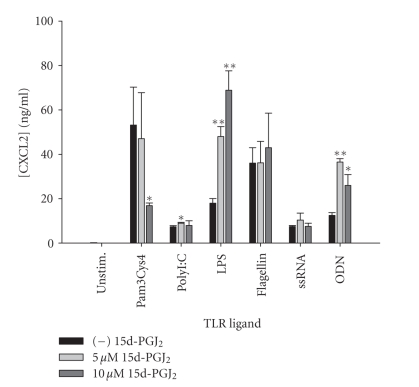
*15d-PGJ_2_ demonstrates differential effects on CXCL2 production by microglia, which are TLR ligand-dependent.* Primary microglia were plated at 2 × 10^5^ cells/well in 96-well plates and incubated overnight. The following day, cells were pretreated for 1 hour with the indicated concentrations of 15d-PGJ_2_ prior to the addition of TLR ligands. Cell-conditioned supernatants were collected at 24 hours following TLR ligand exposure, whereupon CXCL2 levels were determined by ELISA. Significant differences between microglia treated with TLR ligands alone versus TLR ligands + 15d-PGJ_2_ are noted with asterisks (*P < .05;**P < .001). Results presented are representative of two independent experiments.

**Figure 7:**
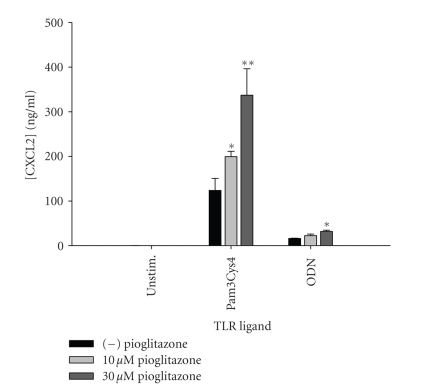
*Pioglitazone augments microglial CXCL2 production in response to Pam3Cys4 and ODN.* Primary microglia were plated at 2 × 10^5^ cells/well in 96-well plates and incubated overnight. The following day, cells were pretreated for 1 hour with the indicated concentrations of pioglitazone prior to the addition of Pam3Cys4 or ODN. Cell-conditioned supernatants were collected at 24 hours following TLR ligand exposure, whereupon CXCL2 levels were determined by ELISA. Significant differences between microglia treated with TLR ligands alone versus TLR ligands + pioglitazone are noted with asterisks (*P < .05;**P < .001). Results presented are representative of two independent experiments.

Chemokines are small molecular weight (8–14 kDa) chemotactic cytokines that are produced locally at sites of inflammation and establish a concentration gradient resulting in the active recruitment of target cell populations [47]. Chemokines are a structurally and functionally related family of proteins subdivided into four groups based on the relative position of conserved N-terminal cysteine residues [47–49]. In general, the chemokine subfamilies show similar, often overlapping specificity with regards to the movements of the target cell populations they orchestrate. One key chemokine involved in the recruitment of neutrophils into areas of inflammation, including the CNS, is CXCL2 (MIP-2) [50–52]. The effects of 15d-PGJ_2_ on microglial CXCL2 expression were complex and varied with each TLR ligand. Specifically, CXCL2 release was either enhanced (polyI:C, LPS, and ODN), reduced (Pam3Cys4), or remained unchanged (flagellin and ssRNA) (Figure 6). Increases in CXCL2 production were also observed following pioglitazone treatment in Pam3Cys- and ODN-stimulated microglia (Figure 7). The overall stimulatory activity of 15d-PGJ_2_ on CXCL2 production is in agreement with reports from other groups [53, 54].

**Figure 8:**
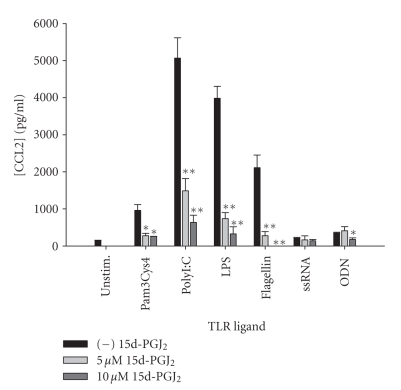
*15d-PGJ_2_ is a potent inhibitor of CCL2 release by microglia in response to a wide range of TLR ligands.* Primary microglia were plated at 2 × 10^5^ cells/well in 96-well plates and incubated overnight. The following day, cells were pretreated for 1 hour with the indicated concentrations of 15d-PGJ_2_ prior to the addition of TLR ligands. Cell-conditioned supernatants were collected at 24 hours following TLR ligand exposure, whereupon CCL2 levels were determined by ELISA. Significant differences between microglia treated with TLR ligands alone versus TLR ligands + 15d-PGJ_2_ are noted with asterisks (*P < .05;**P < .001). Results presented are representative of two independent experiments.

Another chemokine that is associated with mononuclear cell infiltration during various CNS infections is CCL2 (MCP-1), which targets monocyte and lymphocyte entry [52, 55, 56]. Unlike CXCL2, which was differentially regulated by 15d-PGJ_2_ in response to diverse TLR ligands, CCL2 production was uniformly and potently attenuated by this PPAR-*γ* agonist in response to the full array of TLR ligands tested (Figure 8). Similar to IL-12 p40 production, the synthetic TZDs demonstrated differential effects on CCL2 release from TLR ligand activated microglia. Specifically, rosiglitazone was fairly comprehensive in its ability to attenuate CCL2 production with significant reductions observed in response to Pam3Cys4, polyI:C, LPS, flagellin, and ODN, whereas the other TZDs tested (ciglitazone and pioglitazone) did not have much effect on CCL2 release in response to the majority of TLR ligands tested (data not shown). In summary, these results reveal that PPAR-*γ* agonists display a wide range of effects on chemokine production by microglia elicited by TLR ligands.

### 3.3. Effects of PPAR-*γ* agonists on astrocytic proinflammatory mediator production in response to TLR ligands

**Figure 9:**
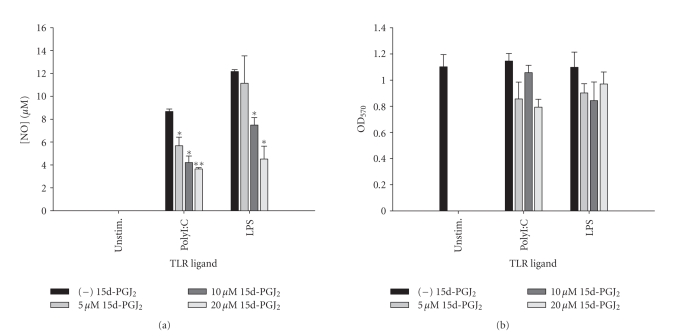
*15d-PGJ_2_ attenuates NO production by astrocytes in response to polyI:C and LPS stimulation.* Primary astrocytes were plated at 1 × 10^5^ cells/well in 96-well plates and incubated overnight. The following day, cells were pretreated for 1 hour with the indicated concentrations of 15d-PGJ_2_ prior to the addition of polyI:C or LPS. Cell-conditioned supernatants were collected at 24 hours following TLR ligand exposure, whereupon NO levels were determined by the Griess reagent (a). The effects of 15d-PGJ_2_ on astrocyte viability were assessed using an MTT assay (b). Significant differences between astrocytes treated with TLR ligands alone versus TLR ligands + 15d-PGJ_2_ are noted with asterisks (*P < .05;**P < .001). Results presented are representative of two independent experiments.

Astrocytes participate in CNS innate immune responses as evident by their ability to produce a wide array of inflammatory mediators in response to diverse stimuli [1, 4]. As already mentioned, these molecules can have dramatic consequences on CNS infection and tissue damage with net effects dictated by factors such as timing and duration of release. To determine the effects of PPAR-*γ* agonists on astrocyte responses to TLR ligands, we examined the production of two proinflammatory mediators produced by activated astrocytes, namely, NO and IL-12 p40. Both polyI:C and LPS were capable of reproducibly inducing NO expression in astrocytes as previously described [31, 57], which was attenuated by 15d-PGJ_2_ in a dose-dependent manner (Figure 9). Similar inhibitory effects on astrocytic NO release in response to polyI:C and LPS were observed with troglitazone and ciglitazone, whereas rosiglitazone and pioglitazone did not modulate NO production (data not shown). Of note was the fact that unlike microglia, which exhibited significant cell death in response to the highest dose of 15d-PGJ_2_ tested (i.e., 20 *μ*M), astrocyte viability was not adversely affected by 15d-PGJ_2_ at any of the concentrations examined. This finding is in agreement with previous reports demonstrating that, in general, primary astrocytes are more refractory to the toxic effects of PPAR-*γ* agonists compared to primary microglia [23, 24, 27].

**Figure 10:**
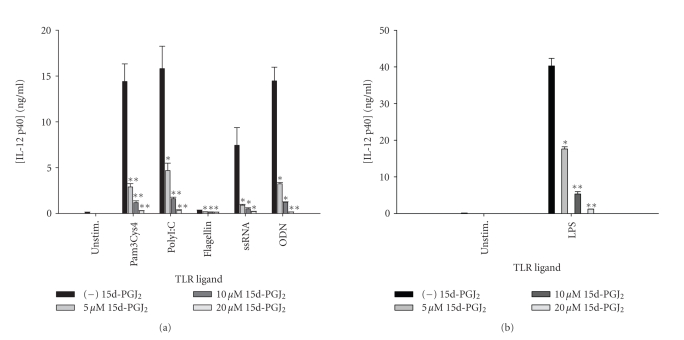
*15d-PGJ_2_ is a global inhibitor of astrocytic IL-12 p40 release following TLR ligand exposure.* Primary astrocytes were plated at 1 × 10^5^ cells/well in 96-well plates and incubated overnight. The following day, cells were pretreated for 1 hour with the indicated concentrations of 15d-PGJ_2_ prior to the addition of TLR ligands. Cell-conditioned supernatants were collected at 24 hours following TLR ligand exposure, whereupon IL-12 p40 levels were determined by ELISA (a) and (b). Significant differences between astrocytes treated with TLR ligands alone versus TLR ligands + 15d-PGJ_2_ are noted with asterisks (*P < .05;**P < .001). Results presented are representative of two independent experiments.

**Figure 11:**
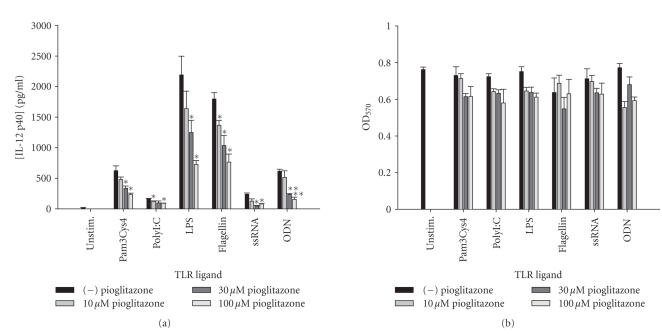
*The TZD pioglitazone inhibits astrocytic IL-12 p40 expression in response to diverse TLR ligands.* Primary astrocytes were plated at 1 × 10^5^ cells/well in 96-well plates and incubated overnight. The following day, cells were pretreated for 1 hour with the indicated concentrations of pioglitazone prior to the addition of TLR ligands. Cell-conditioned supernatants were collected at 24 hours following TLR ligand exposure, whereupon IL-12 p40 levels were determined by ELISA (a). The effects of pioglitazone on astrocyte viability were assessed using an MTT assay (b). Significant differences between astrocytes treated with TLR ligands alone versus TLR ligands + pioglitazone are noted with asterisks (*P < .05;**P < .001). Results presented are representative of two independent experiments.

Similar to the findings obtained with microglia, 15d-PGJ_2_ was a universal and potent inhibitor of IL-12 p40 production by astrocytes in response to all TLR ligands tested (Figure 10). In addition, both pioglitazone and troglitazone were capable of attenuating IL-12 p40 expression in response to the full array of TLR ligands examined (Figure 11 and data not shown), whereas the effects of ciglitazone were variable.

### 3.4. PPAR-*γ* agonists modulate chemokine production by astrocytes following TLR ligand exposure

**Figure 12:**
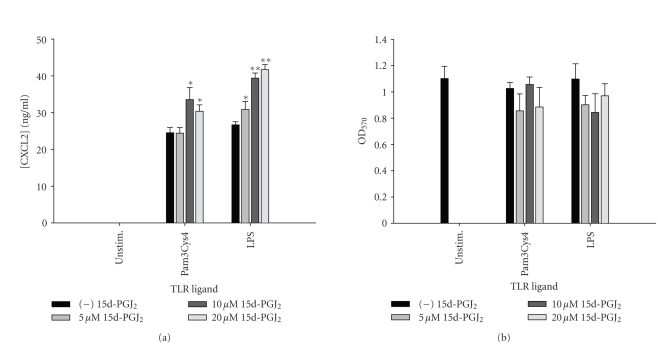
*CXCL2 release is augmented in astrocytes by 15d-PGJ_2_ in response to distinct TLR ligands.* Primary astrocytes were plated at 1 × 10^5^ cells/well in 96-well plates and incubated overnight. The following day, cells were pretreated for 1 hour with the indicated concentrations of 15d-PGJ_2_ prior to the addition of Pam3Cys4 or LPS. Cell-conditioned supernatants were collected at 24 hours following TLR ligand exposure, whereupon CXCL2 levels were determined by ELISA (a). The effects of 15d-PGJ_2_ on astrocyte viability were assessed using an MTT assay (b). Significant differences between astrocytes treated with TLR ligands alone versus TLR ligands + 15d-PGJ_2_ are noted with asterisks (*P < .05;**P < .001). Results presented are representative of two independent experiments.

**Figure 13:**
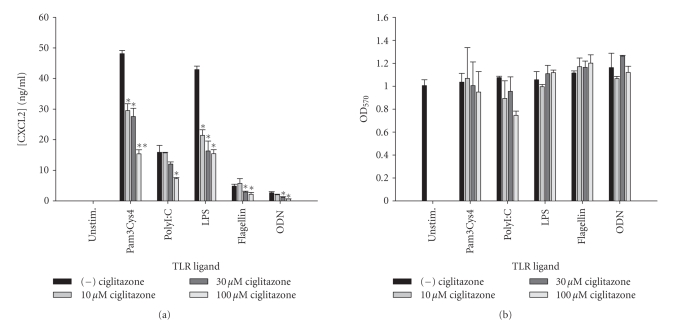
*Ciglitazone attenuates astrocytic CXCL2 expression in response to several TLR ligands.* Primary astrocytes were plated at 1 × 10^5^ cells/well in 96-well plates and incubated overnight. The following day, cells were pretreated for 1 hour with the indicated concentrations of ciglitazone prior to the addition of TLR ligands. Cell-conditioned supernatants were collected at 24 hours following TLR ligand exposure, whereupon CXCL2 levels were determined by ELISA (a). The effects of ciglitazone on astrocyte viability were assessed using an MTT assay (b). Significant differences between astrocytes treated with TLR ligands alone versus TLR ligands + ciglitazone are noted with asterisks (*P < .05;**P < .001). Results presented are representative of two independent experiments.

**Figure 14:**
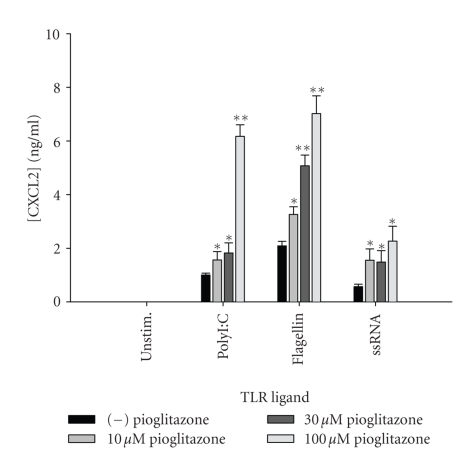
*Pioglitazone enhances CXCL2 release by astrocytes following TLR ligand exposure.* Primary astrocytes were plated at 1 × 10^5^ cells/well in 96-well plates and incubated overnight. The following day, cells were pretreated for 1 hour with the indicated concentrations of pioglitazone prior to the addition of polyI:C, flagellin, or ssRNA. Cell-conditioned supernatants were collected at 24 hours following TLR ligand exposure, whereupon CXCL2 levels were determined by ELISA. Significant differences between astrocytes treated with TLR ligands alone versus TLR ligands + pioglitazone are noted with asterisks (*P < .05;**P < .001). Results presented are representative of two independent experiments.

Although astrocytes are capable of releasing cytokines in response to diverse antigens, they are often considered the major source of chemokines during CNS inflammation [1, 4]. Similar to our recent report, 15d-PGJ_2_ slightly augmented CXCL2 production by astrocytes in response to several TLR ligands examined, namely Pam3Cys4, PGN, and LPS (Figure 12 and data not shown) [24]. Similar increases in CXCL2 release following PPAR-*γ* agonist exposure have also been reported by others [53, 54]. In contrast, each synthetic TZD appeared to differentially regulate CXCL2 release. For example, ciglitazone inhibited CXCL2 expression in response to the entire battery of TLR ligands examined (Figure 13). Conversely, pioglitazone significantly augmented CXCL2 expression in response to polyI:C, flagellin, and ssRNA particularly at the highest dose examined (i.e., 100 *μ*M; Figure 14), whereas rosiglitazone did not dramatically alter CXCL2 levels response to any of the TLR ligands tested in astrocytes (data not shown).

**Figure 15:**
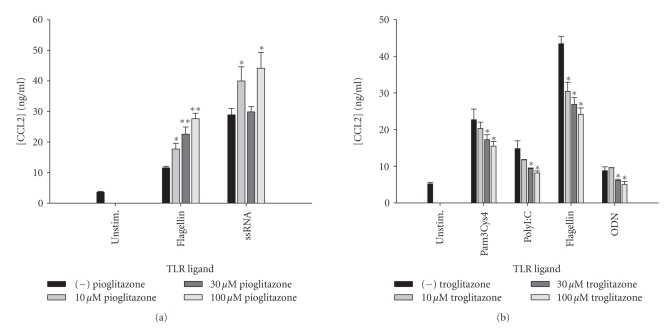
*TZDs exert differential effects on astrocytic CCL2 production following TLR ligand treatment.* Primary astrocytes were plated at 1 × 10^5^ cells/well in 96-well plates and incubated overnight. The following day, cells were pretreated for 1 hour with the indicated concentrations of pioglitazone (a) or troglitazone, (b) prior to the addition of TLR ligands. Cell-conditioned supernatants were collected at 24 hours following TLR ligand exposure, whereupon CCL2 levels were determined by ELISA (a) and (b). Significant differences between astrocytes treated with TLR ligands alone versus TLR ligands + TZDs are noted with asterisks (*P < .05;**P < .001). Results presented are representative of two independent experiments.

Similar to CXCL2, pioglitazone treatment increased CCL2 production in response to flagellin, and ssRNA primarily at the highest dose tested, whereas troglitazone led to significant reductions in CCL2 release following Pam3Cys4, polyI:C, flagellin, and ODN treatment (Figure 15). In general, ciglitazone and rosiglitazone had little effect on CCL2 production by astrocytes in response to the majority of TLR ligands tested (data not shown). These results indicate that despite their inclusion within the same family, specific PPAR-*γ* agonists differentially target proinflammatory genes in distinct manners.

## 4. DISCUSSION

In order for the CNS to respond to infectious insults, a rapid and efficient host immune response must be initiated and directed to expedite pathogen elimination. One means to achieve this goal is through the triggering of TLRs expressed on resident glia that signal proinflammatory mediator release in an attempt to quell infection [11, 12]. However, recent evidence also suggests that these normally protective immune responses can become dysregulated, culminating in the destruction of surrounding normal CNS parenchyma [13]. Therefore, fine tuning the resultant immune response to achieve maximal pathogen clearance concomitant with minimal tissue damage would represent a best case scenario for the management of a wide array of CNS infectious diseases including bacterial meningitis, HIVE, brain abscess, and other viral infections. The purpose of this study was to perform a comprehensive analysis of the ability of several PPAR-*γ* agonists to regulate glial activation in response to a panel of TLR ligands that may be encountered during native CNS infections. It is envisioned that this information may be exploited as a first step towards the derivation of specific treatment strategies that could be implemented with existing therapies for CNS infections to maximize benefit to patients.

One obvious distinction between the various PPAR-*γ* agonists tested to modulate TLR-dependent glial activation was the finding that 15d-PGJ_2_ consistently led to more dramatic and widespread decreases in inflammatory mediator production compared to the synthetic TZDs. This relationship was observed with both primary microglia and astrocytes and is in agreement with previous reports by us and other laboratories where 15d-PGJ_2_ exerted potent inhibitory effects at lower effective concentrations compared to TZDs, despite the fact that the former exhibits a lower binding affinity to PPAR-*γ* [26, 27, 58, 59]. In addition, differences were observed between the immune modulatory effects within the group of TZDs tested. This was somewhat surprising; however, Storer et al. also reported that ciglitazone and pioglitazone had no effect on TNF-*α* or CCL2 production by microglia in response to LPS [27], similar to our findings in the present study. However, a few discrepancies between these reports also exist, which might be explained by the fact that only a single TLR ligand was examined (i.e., LPS) and the concentrations of several TZDs exceeded the maximal dose tested in the current study. In general, only one TZD, rosiglitazone, exerted rather global effects on the inflammatory mediators examined here, whereas the other compounds (i.e., ciglitazone, troglitazone, and pioglitazone) demonstrated differential effects that were dependent on the TLR ligand as well as the proinflammatory mediator measured. The reason responsible for these differences is not clear but distinct chemical structures of the various TZDs have been suggested to contribute to their unique properties [60]. The widespread effects of rosiglitazone on glial inflammatory mediator production are in agreement with the fact that this TZD has been reported to impact numerous PPAR-*γ* isoforms including PPAR-*γ*1 and PPAR-*γ*2, whereas other TZDs have been reported to selectively target a single PPAR-*γ* subtype [61, 62]. Other studies have revealed distinct differences in the effectiveness of TZD members in regulating changes in glucose metabolism in astrocytes [63] and mitochondrial function [64]. In addition, the EC_50_ of individual TZD compounds for PPAR-*γ* does vary within this group of molecules depending on the experimental readout examined, which may contribute to their differential effects [61, 62]. Alternatively, studies have shown that the coactivator proteins interacting with PPAR-*γ* differ in a ligand-dependent manner [65]. Unfortunately, there are few reports where comprehensive side-by-side comparisons have been made with regard to the effects of various TZD compounds on neuroinflammation either in vitro or in vivo. Making direct comparisons between various TZDs in disparate models of neuroinflammation should be viewed cautiously since differences in disease models, inflammatory stimuli, and species examined all have the potential to influence the results obtained.

Our findings demonstrating the inhibitory effects of 15d-PGJ_2_ on TLR ligand-induced glial activation are similar to those observed following LPS stimulation. Specifically, 15d-PGJ_2_ has been shown to inhibit LPS-induced NO [27–29, 66], TNF-*α* [27, 28, 66], and IL-12 family member expression [26] in microglia and/or astrocytes. Importantly, this report has provided a more comprehensive analysis of the effects of PPAR-*γ* agonists on glial activation with the inclusion of a battery of TLR ligands that would be encountered during various CNS infectious diseases. Earlier studies have primarily focused on the immune modulatory effects of PPAR-*γ* agonists in response to the TLR4 ligand LPS, and therefore, this study is also novel from the perspective that numerous TLR ligands were evaluated.

Interestingly, both 15d-PGJ_2_ and pioglitazone were found to augment CXCL2 release by microglia and astrocytes in response to specific TLR ligands. In particular, 15d-PGJ_2_ enhanced CXCL2 production in response to LPS stimulation in both microglia and astrocytes, in agreement with previous reports [53, 54]. In addition, recent results from our laboratory have revealed that CXCL2 release was slightly increased by 15d-PGJ_2_ and downregulated by ciglitazone in response to the gram-positive pathogen *S. aureus* in primary astrocytes [24], in corroboration with the current report.

Elevated levels of endogenous 15d-PGJ_2_ have been associated with the resolution of inflammation in vivo, suggesting that it functions as negative feedback regulator of inflammatory responses [67, 68]. This study demonstrates that 15d-PGJ_2_ is an effective and selective inhibitor of glial activation in response to TLR ligands, suggesting that it may be capable of modulating chronic microglial and astrocyte responses during the course of CNS infectious diseases. Evidence to support this concept is provided by our findings that 15d-PGJ_2_ effectively inhibited IL-12 p40 and CCL2 expression in microglia and astrocytes, two molecules with important functions in the differentiation of CD4^+^ Th1 cells and monocyte and T cell influx into the infected CNS [52, 55, 56, 69, 70]. It is possible that the downregulation of these mediators in microglia and astrocytes following 15d-PGJ_2_ treatment results in the failure to recruit and/or stimulate Ag-specific T cells in the CNS parenchyma. Therefore, preventing chronic microglial activation by 15d-PGJ_2_ or synthetic PPAR-*γ* agonists may help to resolve inflammation earlier, resulting in less damage to surrounding normal brain parenchyma.

In summary, these studies demonstrate that not all PPAR-*γ* agonists are created equal in terms of their ability to modulate proinflammatory mediator release by activated microglia and astrocytes. Specifically, differences were specific to the type of TLR ligand examined. When considering the potential utility of PPAR-*γ* agonists for modulating pathological inflammation typical of several CNS infectious diseases, critical issues such as the timing and length of PPAR-*γ* administration and doses of compound must be considered. For example, it appears likely that compounds should be delivered at periods at or nearing pathogen clearance since a significant attenuation of the host immune response would be counterproductive to infection resolution. Upon pathogen elimination, PPAR-*γ* agonists may minimize damage to surrounding tissue by downregulating exaggerated CNS immune responses that could be perpetuated by microbial debris (i.e., cell wall fragments such as LPS and PGN or pathogen nucleic acids) via continued engagement of TLRs. Indeed, recent studies by our laboratory have revealed that the PPAR-*γ* agonist ciglitazone demonstrates beneficial effects with delayed administration in an experimental brain abscess model as revealed by accelerated abscess encapsulation and reduction in bacterial burdens [71]. In addition, recent studies by other groups have revealed beneficial effects of PPAR-*γ* agonists in other infectious disease paradigms [72–76]. Therefore, the current study can be used as a guide to facilitate the selection of PPAR-*γ* agonists as candidates for intervention during CNS infectious diseases.

## Figures and Tables

**Table 1:** Summary of PPAR-*γ* effects on PAMP-activated microglia reported in this study.

TLR ligand						
PPAR-*γ* agonist	Pam3Cys	polyI:C	LPS	Flagellin	ssRNA	ODN
15d-PGJ_2_	↓ IL-12	↓ IL-12	↓ IL-12	↓ IL-12	↓ IL-12	↓ IL-12
	↓ TNF-*α*	↓ TNF-*α*	↓ TNF-*α*	↓ TNF-*α*	TNF-*α* ND*	↓ TNF-*α*
	↓ CXCL2	↑ CXCL2	↑ CXCL2	No effect CXCL2	No effect CXCL2	↑ CXCL2
	↓ CCL2	↓ CCL2	↓ CCL2	↓ CCL2	No effect CCL2	↓ CCL2

Rosiglitazone	↓ IL-12	↓ IL-12	↓ IL-12	↓ IL-12	↓ IL-12	↓ IL-12
	↓ TNF-*α*	↓ TNF-*α*	No effect TNF-*α*	↓ TNF-*α*	TNF-*α* ND	No effect TNF-*α*

Pioglitazone	↓ IL-12	↓ IL-12	↓ IL-12	↓ IL-12	↓ IL-12	↓ IL-12
	↑ CXCL2					↑ CXCL2

*ND; not detectable.

**Table 2:** Summary of PPAR-*γ* effects on PAMP-activated astrocytes reported in this study.

TLR ligand						
PPAR-*γ* agonist	Pam3Cys	polyI:C	LPS	Flagellin	ssRNA	ODN
	↓ IL-12	↓ IL-12	↓ IL-12	↓ IL-12	↓ IL-12	↓ IL-12
15d-PGJ_2_	↑ CXCL2	↓ NO	↓ NO			
			↑ CXCL2			

Ciglitazone	↓ CXCL2	↓ CXCL2	↓ CXCL2	↓ CXCL2	NR*	↓ CXCL2

Pioglitazone	↓ IL-12	↓ IL-12	↓ IL-12	↓ IL-12	↓ IL-12	↓ IL-12
	↑ CXCL2			↑ CXCL2	↑ CXCL2	
				↑ CCL2	↑ CCL2	

Troglitazone	↓ CCL2	↓ CCL2	NR	↓ CCL2	NR	↓ CCL2

*NR: not reported.
